# Reliability Risk Mitigation in Advanced Packages by Aging-Induced Precipitation of Bi in Water-Quenched Sn–Ag–Cu–Bi Solder

**DOI:** 10.3390/ma17143602

**Published:** 2024-07-21

**Authors:** Vishnu Shukla, Omar Ahmed, Peng Su, Tengfei Jiang

**Affiliations:** 1 Department of Materials Science and Engineering, Advanced Materials Processing and Analysis Center, University of Central Florida, Orlando, FL 32816, USA; vishnu.raj.shukla@ucf.edu; 2 Juniper Networks, Sunnyvale, CA 94089, USA

**Keywords:** lead-free solder, SAC+X, solder interconnects, quasistatic nanoindentation, continuous stiffness measurement, intermetallic compound, aging, precipitation strengthening, solid-solution strengthening

## Abstract

Bi-doped Sn–Ag–Cu (SAC) microelectronic solder is gaining attention for its utility as a material for solder joints that connect substrates to printed circuit boards (PCB) in future advanced packages, as Bi-doped SAC is reported to have a lower melting temperature, higher strength, higher wettability on conducting pads, and lower intermetallic compound (IMC) formation at the solder-pad interface. As solder joints are subjected to aging during their service life, an investigation of aging-induced changes in the microstructure and mechanical properties of the solder alloy is needed before its wider acceptance in advanced packages. This study focuses on the effects of 1 to 3 wt.% Bi doping in an Sn–3.0Ag–0.5Cu (SAC305) solder alloy on aging-induced changes in hardness and creep resistance for samples prepared by high cooling rates (>5 °C/s). The specimens were aged at ambient and elevated temperatures for up to 90 days and subjected to quasistatic nanoindentation to determine hardness and nanoscale dynamic nanoindentation to determine creep behavior. The microstructural evolution was investigated with a scanning electron microscope in tandem with energy-dispersive spectroscopy to correlate with aging-induced property changes. The hardness and creep strength of the samples were found to increase as the Bi content increased. Moreover, the hardness and creep strength of the 0–1 wt.% Bi-doped SAC305 was significantly reduced with aging, while that of the 2–3 wt.% Bi-doped SAC305 increased with aging. The changes in these properties with aging were correlated to the interplay of multiple hardening and softening mechanisms. In particular, for 2–3 wt.% Bi, the enhanced performance was attributed to the potential formation of additional Ag_3_Sn IMCs with aging due to non-equilibrium solidification and the more uniform distribution of Bi precipitates. The observations that 2–3 wt.% Bi enhances the hardness and creep strength of the SAC305 alloy with isothermal aging to mitigate reliability risks is relevant for solder samples prepared using high cooling rates.

## 1. Introduction

Lead-free solder alloys are widely used in microelectronics packaging since Sn–Pb solder was banned for usage in electronic devices due to its toxicity [1,2,3]. A variety of Sn-based solder materials have since been investigated for their manufacturability, reliability, and performance, all of which determine their suitability for microelectronics packages [4,5,6,7,8,9,10,11]. 96.5Sn–3.0Ag–0.5Cu (SAC305) was found to be a suitable composition for ball grid array (BGA) solder joints due to its low cost, good solderability, compatibility with multiple flux types, good wettability, and desirable thermomechanical reliability performance [8,12,13,14,15,16,17]. However, future advanced applications, such as 5G and high-performance computing (HPC), are likely to involve larger components and thicker printed circuit boards (PCBs), which will result in a higher mechanical loading on solder interconnects, necessitating new solder alloys with superior properties such as better creep strength, fatigue resistance, and hardness. Additionally, the service life of solder joints involves aging and thermal cycling, which leads to various microstructural changes, including changes to the intermetallic compound (IMC) size and distribution changes [18,19,20,21]. These aging-induced changes result in corresponding changes in the mechanical properties such as hardness and creep strength [20,21]. Therefore, it is also important to investigate the property changes that occur in new solder alloys with aging to examine the underlying microstructural causations of those property changes and the effects of alloying elements.

Various studies suggest that Bi-doped Sn–Ag–Cu (SAC) solder could be a promising candidate for future packaging applications. Chantaramanee et al. reported that 2 wt.% Bi doping in SAC305 increased the ultimate tensile strength and microhardness of SAC305 by 57% [22]. In the same study, SAC305–Bi/Cu joints were found to have a thinner IMC layer along with a higher shear strength as compared to SAC305/Cu joints. Ahmed et. al. performed uniaxial tensile tests on SAC305 and SAC305 doped with 3.3 wt.% Bi and reported large aging-induced degradations in the tensile strength of SAC305 while SAC305 doped with 3.3 wt.% Bi did not show any degradation in tensile strength with aging [23]. Athamneh et. al., in their study on the fatigue behavior and shear strength of SAC–Bi reported a much higher fatigue life and shear strength in comparison to SAC305 [24]. Additionally, doping Bi in microelectronic solder is known to increase its strength [25,26,27,28], suppress the intermetallic compound (IMC) growth kinetics [29,30,31], improve the wettability between the solder and pad [28,32,33], and lower the melting temperature of the solder [32,34,35], all of which are expected to improve the reliability of solder joints. The performance attributes of SAC–Bi necessitate a comprehensive study of one of the most widely used microelectronic solder alloys, i.e., SAC305, with varying Bi contents. Moreover, aging-induced changes in the microstructure and properties of the alloy must be revealed to elucidate the role played by Bi in the microstructural and property changes.

The microstructure and, hence, the properties of the SAC–Bi solder alloy are also expected to vary with changes in sample preparation techniques and various process parameters such as the cooling rate. Investigations conducted by various authors indicate that variations in the microstructures of SAC–Bi alloys are possible when there are minor variations in process parameters. It has been reported that SAC305–3Bi samples prepared in glass molds using a reflow cooling rate of 3 °C/s have the presence of Bi precipitates in an as-solidified condition [25,36]. In the same investigations, these precipitates were reported to dissolve in the Sn matrix with high-temperature aging, owing to the higher solubility of Bi in Sn at higher temperatures. However, in another study, an absence of Bi precipitates in SAC305–4Bi solder joints in an as-solidified condition was reported when the same cooling rate was used [37]. Moreover, in contrast to the findings on aging by Ahmed et al. and Belhadi et al. [25,36], Bi precipitates were reported after the high-temperature aging of SAC–Bi alloys in other studies [38,39]. This calls for further investigation on the aging-induced changes brought about by Bi doping in SAC solder specimens prepared using different process parameters. In addition, the cooling rate of solder specimens during solidification is known to affect the microstructure and properties of the samples. For SAC solder, studies have shown that a higher cooling rate promotes non-equilibrium solidification and results in higher fractions of β-Sn dendrites, with reduced secondary dendrite arm spacing [40,41,42]. As the cooling rate increases, more finely and uniformly dispersed Ag_3_Sn IMC was also observed [41,43,44]. Increased microhardness has also been observed for SAC305 subjected to a higher cooling rate [40,43,44], although such studies are not available for SAC305+Bi. 

This paper presents a detailed study on SAC305 doped with 1, 2 and 3 wt.% Bi that was conducted to investigate the effect of Bi on the changes in hardness and creep strength when the samples were subjected to isothermal aging. The samples were prepared by vacuum casting followed by water quenching, resulting in a cooling rate that was within the range of, but on the high end of, the cooling rate recommended by the JEDEC standard [45]. The isothermal aging involved temperatures of 25 °C, 75 °C, and 125 °C, covering a large spectrum of service temperatures of a solder joint. The aging-induced changes in hardness and creep strength were quantified after aging durations of 30, 60, and 90 days, using nanoindentation. Hardness and stiffness from micron-sized regions within the samples were obtained to quantify the mechanical properties of a micron-sized specimen. The creep behavior was extracted using continuous measurement of stiffness (CMS) methods. The various phases present, and the size and distribution of the Bi precipitates and IMCs, were identified with scanning electron microscopy (SEM) and energy-dispersive spectroscopy (EDS). Together, the microstructural causations of the corresponding property changes with aging were determined and were found to correlate to each other. In particular, the roles of Bi solute atoms and Bi precipitates on the microstructure and property changes were revealed and discussed.

## 2. Materials and Methods

A. Sample Preparation

Solder samples used in this study were made by vacuum casting [46]. Briefly, solder bars of four compositions, i.e., Sn–3.0Ag–0.5Cu (SAC305), Sn–3.0Ag–0.5Cu–1Bi (SAC305+1% Bi), Sn–3.0Ag–0.5Cu–2Bi (SAC305+2% Bi) and Sn–3.0Ag–0.5Cu–3Bi (SAC305+3% Bi) (ACCURUS SCIENTIFIC Co., Ltd., Tainan, Taiwan) were melted in inert crucibles. The molten solder was suctioned into a glass tube mold that was connected to a vacuum pump. The glass tube was then immediately water-quenched to ensure a uniform microstructure throughout the length of the tube. The dimensions of the casts were 0.5 mm × 0.5 mm, creating solder samples of the same cross-sectional dimensions, which were similar to the dimensions of BGA solder joints. The as-casted samples were cut into 3–5mm lengths suitable for nanoindentation and further characterization.

B. Aging Experiment

For all compositions, specimens were subjected to isothermal aging at three different temperatures, as follows: 25 °C, 75 °C, and 125 °C. For each temperature, the specimens were aged for durations of 30, 60, and 90 days. The aging matrix comprising all the temperature–time combinations is shown in Table 1.

C. Investigation of Microstructures

Specimens subjected to aging were mounted in epoxy, polished, and coated with gold for examination under a scanning electron microscope (Zeiss ULTRA-55 FEG SEM, ZEISS, Oberkochen, Baden-Württemberg, Germany) with energy-dispersive spectroscopy (EDS). Secondary electron (SE) and backscattered electron (BSE) images of the same area within the sample were captured to reveal the morphology and distribution of the phases present, and EDS was used to identify the composition of the phases.

D. Measurement of Hardness and Creep Strength

A Bruker TI Premier nanoindenter (Bruker, Billerica, MA, USA) equipped with a three-sided pyramidal Berkovich tip was used to carry out the nanoindentation measurements. To obtain the hardness of the samples, quasistatic nanoindentation was performed, where the load function consisted of 10 s of loading time, 3 s of dwell at a peak load of 4 mN, and 10 s of unloading time. The test on the samples returned a load (P)–indentation depth (h) curve, from which the hardness (H) of the samples was calculated as H = P/A, where A = 24.5 h^2^ is the projected contact area. Forty evenly spaced indents were made for each sample to ensure that the entire sample cross-section was covered and to obtain values representative of the entire sample.

**Figure 1:**
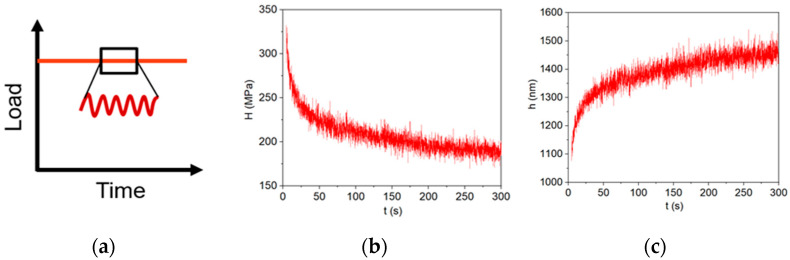
(**a**) Illustration of the nanoDMA load function, where a small sinusoidal load is superimposed on an applied load; (**b**) hardness–time plot for as-cast SAC305; and (**c**) indentation depth–time plot for as-cast SAC305.

To obtain the creep strength, nanoscale dynamic mechanical analysis (nanoDMA), a dynamic nanoindentation technique, was used. In this particular technique, a reference frequency method was employed to correct for thermal drift during the measurement, enabling reliable creep tests at nanoscale [47]. During testing, the indenter tip applied a constant load of 10 mN on the sample for 300 s, with a small sinusoidal load of 0.4 mN superimposed on the constant load. This load function enables continuous measurement of stiffness and hardness to obtain time-dependent material behaviors. Similar techniques have been used to evaluate the creep properties of solder by various researchers [47,48,49]. A schematic of the load function for the dynamic testing is shown in Figure 1a. Representative H–t and h–t plots obtained from the dynamic testing are shown in Figure 1b,c.

**Figure 2:**
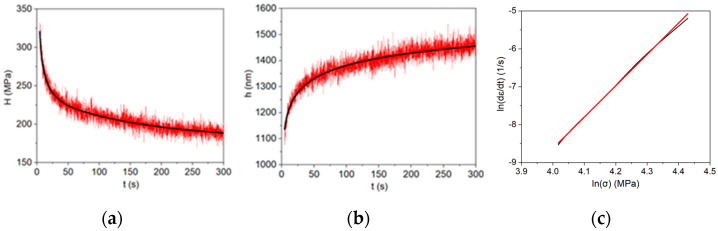
(**a**) Fitted H–t plot for as-cast SAC305; (**b**) fitted h–t plot for as-cast SAC305; and (**c**) log–log plot of creep strain rate ($\dot{\varepsilon }$) vs. stress (σ) for as-cast SAC305.

The obtained H–t and h–t plots were fitted as shown in Figure 2a,b and the fitted data were used to determine the stress (σ) and corresponding strain rate ($\dot{\varepsilon }$) for each of the stress values using the following equations:

(1) $$\sigma =\frac{H}{3}$$

(2) $$\dot{\varepsilon }=\frac{1}{h}\frac{dh}{dt}$$

The creep strain rate vs. stress was plotted in log scale for each nanoDMA test, which fitted in a linear equation, indicating that the samples followed the Norton creep equation, as follows:

(3) $$\dot{\varepsilon }=A\sigma^{n}$$

The fitted linear plot shown in Figure 2c was extrapolated to determine the creep strain rate ($\dot{\varepsilon }$) that corresponded to the selected creep strength (σ) for each sample.

## 3. Results

A. Microstructural Evolution

**Figure 3:**
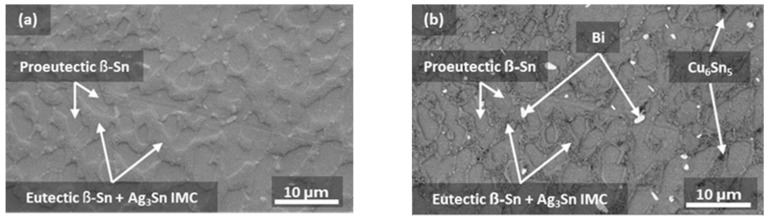
(**a**) SEM–SE micrograph; and (**b**) SEM–BSE micrograph of an SAC305+3% Bi sample aged for 90 days at 125 °C.

An SE micrograph and a BSE micrograph of the same location within an SAC305+3% Bi sample aged for 90 days at 125 °C are shown in Figure 3a and Figure 3b, respectively. The SE micrograph reveals a dendritic microstructure commonly seen in Sn-rich solders, and four phases are clearly distinguished in the BSE micrograph. 

**Figure 4:**
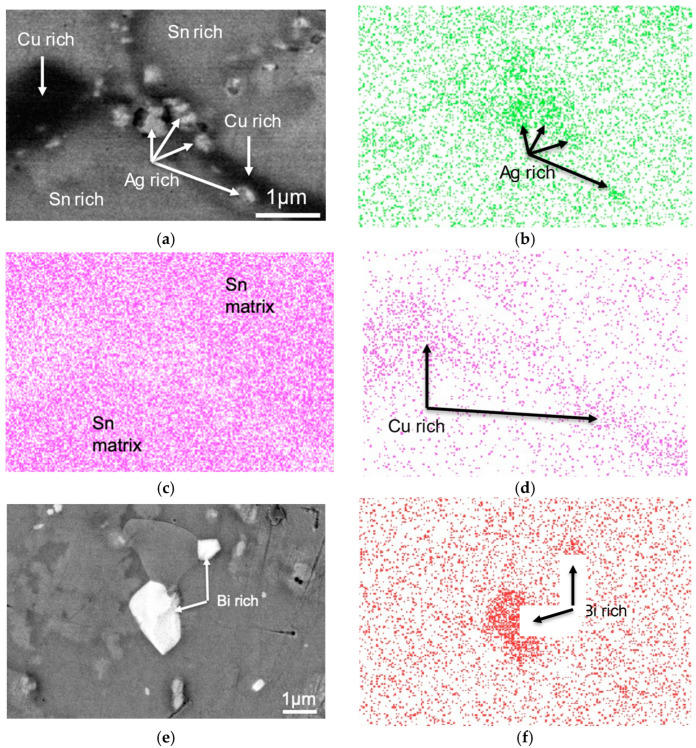
(**a**) SEM–BSE micrograph of a region in an SAC305+3% Bi sample aged for 90 days at 125 °C; (**b**) EDS Ag mapping of the micrograph shown in (**a**); (**c**) EDS Sn mapping of the micrograph shown in (**a**); (**d**) EDS Cu mapping of the micrograph shown in (**a**); (**f**) SEM–BSE micrograph of another region in the SAC305–3% Bi sample aged for 90 days at 125 °C showing a bright white phase, found to be rich in Bi; and (**f**) EDS Bi mapping of the micrograph shown in (**e**).

EDS elemental mapping was carried out on two regions of the sample and the results are shown in Figure 4. Figure 4b shows that the particles in the interdendritic regions are Ag-rich, and, together with the Sn elemental mapping in Figure 4c, indicate the presence of eutectic β-Sn and Ag_3_Sn embedded in the proeutectic β-Sn matrix. The dark phase in the interdendritic region shown in Figure 4d was found to be Cu-rich; based on the Sn–Cu phase diagram, this phase was identified as Cu_6_Sn_5_ IMCs. Figure 4e,f shows another region in the sample that contains bright white particles in the SEM–BSE micrograph. EDS mapping showed that the particles were Bi-rich. Given the limited solubility of between 1–2 wt.% of Bi in Sn [28], these bright particles were identified as Bi precipitates. The identified phases are shown in Figure 3.

**Figure 5:**
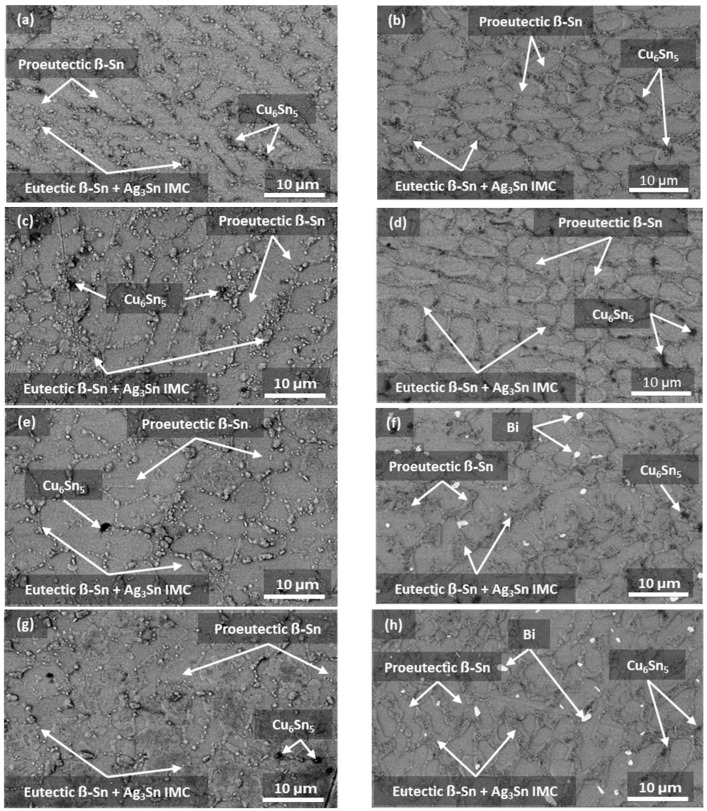
SEM–BSE micrographs: (**a**) as-cast SAC305; (**b**) SAC305 aged at 125 °C for 90 days; (**c**) as-cast SAC305+1% Bi; (**d**) SAC305+1% Bi aged at 125 °C for 90 days; (**e**) as-cast SAC305+2% Bi, (**f**) SAC305+2% Bi aged at 125 °C for 90 days; (**g**) as-cast SAC305+3% Bi; and (**h**) SAC305+3% Bi aged at 125 °C for 90 days.

To reveal the effects of Bi content on the evolution of microstructures under different aging durations and temperatures, the microstructures of SAC305, SAC305+1 wt.% Bi, SAC305+2 wt.% Bi and SAC305+3 wt.% Bi samples after 90 days of aging at 125 °C were compared to their microstructures in the as-cast conditions, as shown in Figure 5. All the as-cast and aged microstructures showed a resemblance to the pseudo-eutectic microstructures of reflowed solder joints of similar sizes [50], where Ag_3_Sn and Cu_6_Sn_5_ IMCs were found to be dispersed in the Sn matrix. Bi precipitates were absent in the as-cast microstructures for all four alloys (Figure 5a,c,e,g). After aging, Bi precipitates were absent in the SAC305 and SAC305+1 wt.% Bi samples (Figure 5b,d) but appeared in the SAC305+2 wt.% Bi and SAC305+3 wt.% Bi samples (Figure 5f,h). 

**Figure 6:**
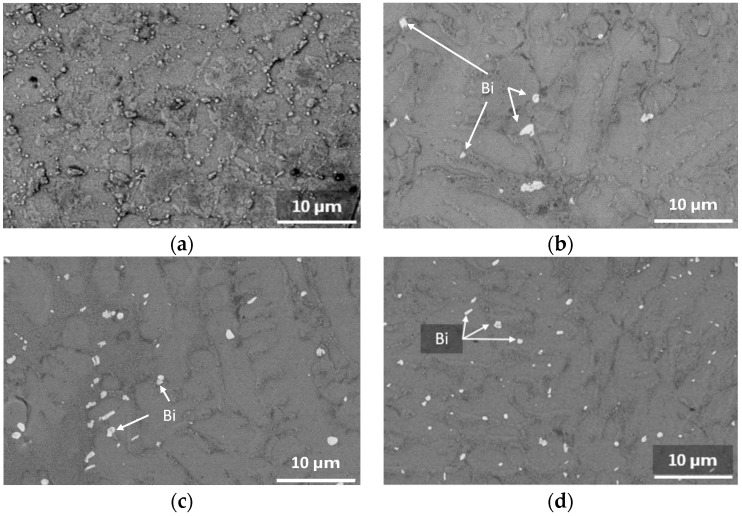
SEM–BSE micrographs: (**a**) as-cast SAC305+3% Bi; (**b**) SAC305+3% Bi aged at 125 °C for 30 days; (**c**) SAC305+3% Bi aged at 125 °C for 60 days; and (**d**) SAC305+3% Bi aged at 125 °C for 90 days.

The evolution of Bi precipitation with aging time is further shown in the BSE micrographs in Figure 6 for SAC305+3% Bi in the as-cast condition and after 30, 60, and 90 days of aging at 125 °C. While absent in the as-cast condition, Bi precipitations were observed in SAC305+3% Bi after aging at 125 °C for 30, 60, and 90 days. Image analysis of the SEM–BSE micrographs of as-cast and aged SAC305+3% Bi samples indicated that the area fraction of Bi precipitates increased from 0% in the as-cast condition to about 1.1% after aging at 125 °C for 30, 60, and 90 days. Although Bi precipitates cover a similar area fraction after aging durations of 30, 60, and 90 days, a comparison of Figure 6b–d showed that the Bi precipitates appeared to be more finely dispersed with increased aging durations.

B. Hardness

**Figure 7:**
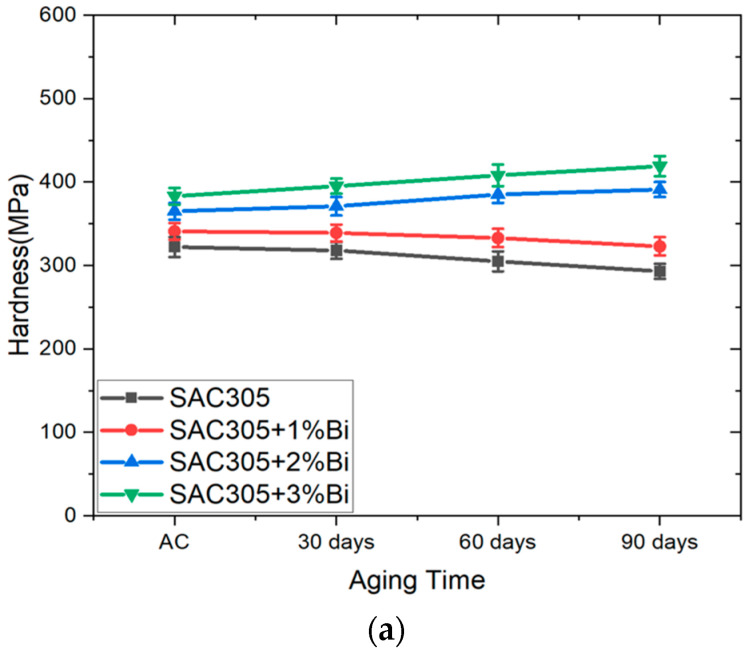

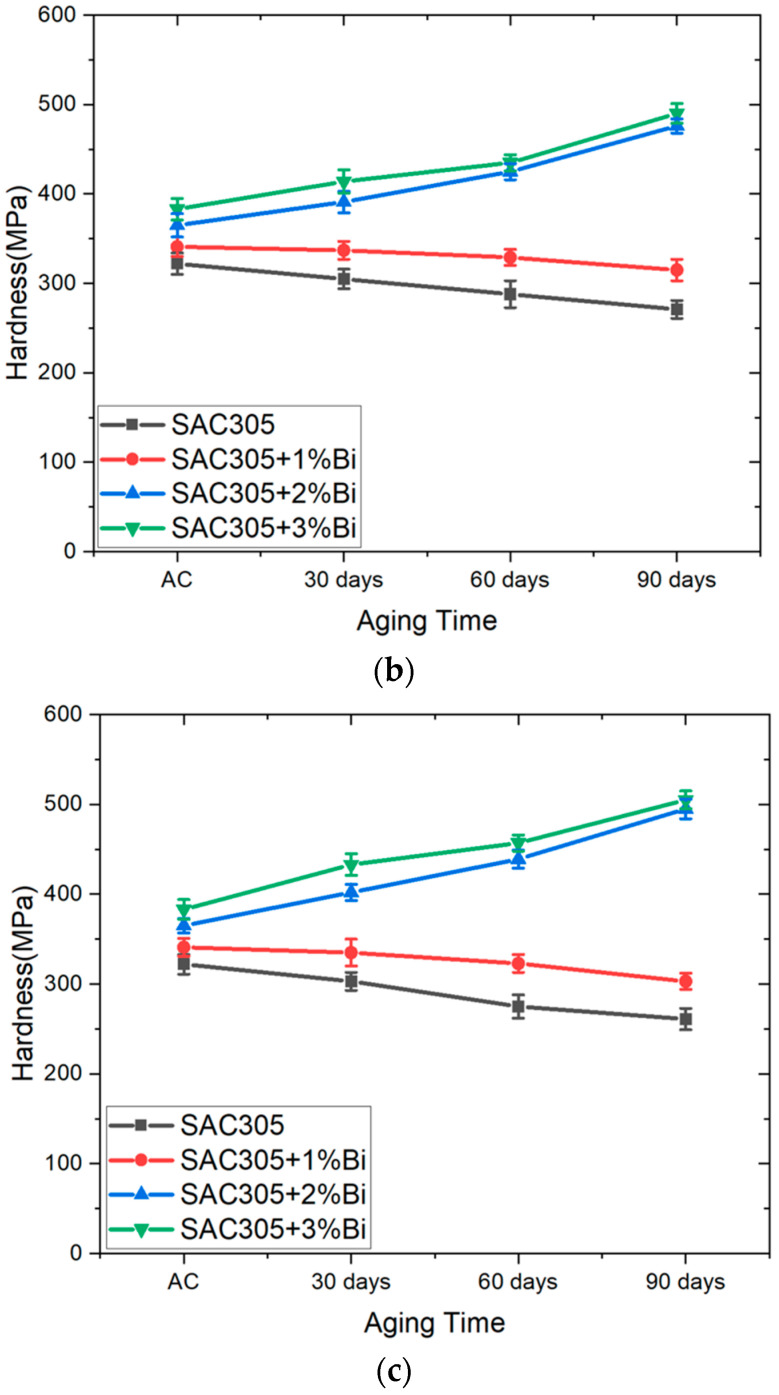
Hardness values of SAC305–xBi (x = 0, 1, 2, 3 wt.%) alloys after aging for 0 days (as-cast), 30 days, 60 days, and 90 days at: (**a**) 25 °C; (**b**) 75 °C; and (**c**) 125 °C.

Hardness values of the as-cast and aged samples obtained from quasistatic nanoindentation are shown in Figure 7. For any given aging condition, including the as-cast state, the hardness of the samples increased with a higher Bi content. However, for each individual alloy composition, aging-induced hardness changes were different. For SAC305 and SAC305 doped with 1 wt.% Bi, the hardness decreased with aging at any aging temperature. As an example, after aging for 90 days at 125 °C, the hardness decreased by 19% for SAC305 and by 11% for SAC305 with 1 wt.% Bi doping compared to the as-cast condition. On the contrary, SAC305 doped with 2 wt.% Bi and 3 wt.% Bi showed increased hardness with isothermal aging for up to 90 days at all aging temperatures. Both 2 and 3 wt.% Bi doping in SAC305 resulted in an increase in hardness of 32% with aging for 90 days at 125 °C, as shown in Figure 7c.

C. Creep Strength

**Figure 8:**
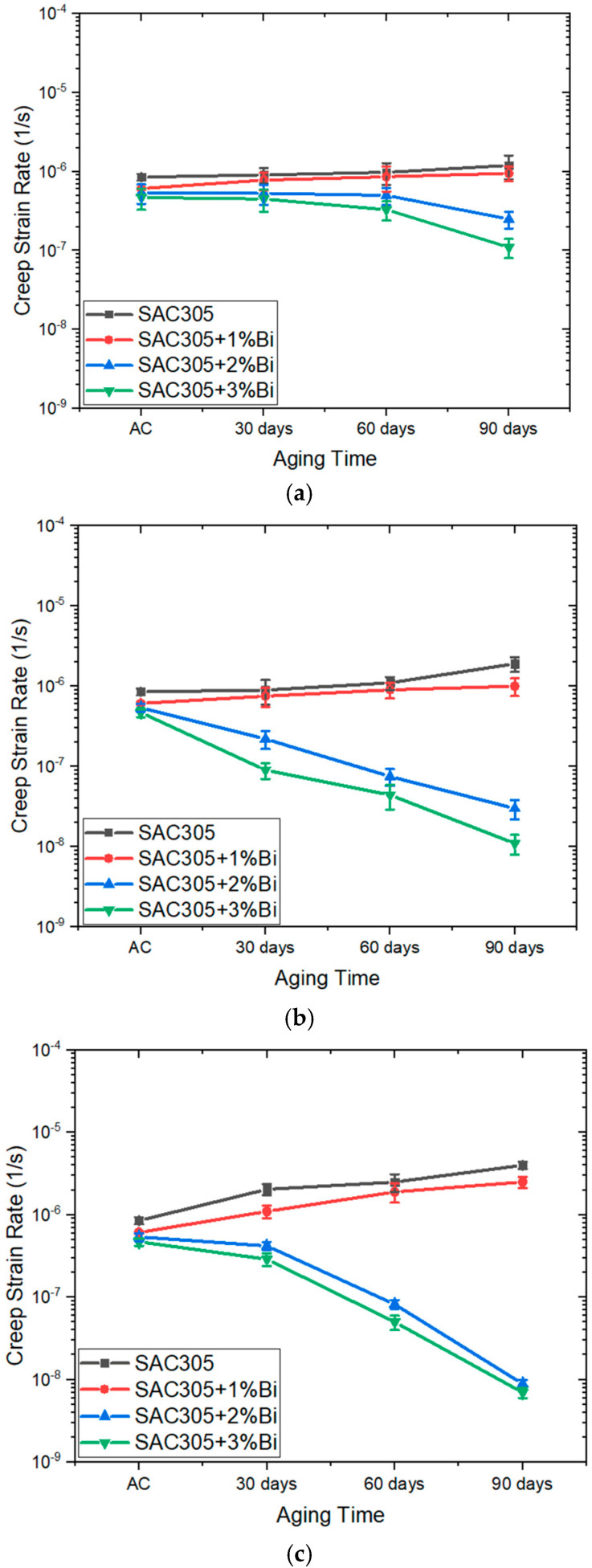
Creep strain rate values at 33 MPa of SAC305–xBi (x = 0, 1, 2, 3 wt.%) alloys after aging for 0 days (as-cast), 30 days, 60 days, and 90 days at: (**a**) 25 °C; (**b**) 75 °C; and (**c**) 125 °C.

For an arbitrarily chosen stress of 33 MPa from among the range of stress values typically experienced by solder balls in packages [51], the creep strain rate was obtained for every sample and was plotted as a function of aging duration for all isothermal aging temperatures (Figure 8). In the as-cast condition, a lower creep strain rate at 33 MPa was observed for a higher Bi content. The creep strain rate at 33 MPa of SAC305+3 wt.% Bi was 55% lower than that of SAC305 in the as-cast condition. Aging at any temperature caused the creep strain rate at 33 MPa to increase for SAC305 and SAC305 with 1 wt.% Bi. As an example, after room-temperature aging at 25 °C for 90 days, the creep rate of SAC305 was found to be 150% of its value in an as-cast condition. The creep rate of SAC305+1 wt.% Bi showed a similar trend, with about a 50% increase in the creep rate value as compared to the as-cast state. In contrast, the creep rates at 33 MPa for SAC305 with 2 wt.% and 3 wt.% Bi were reduced at all temperatures with aging; a 2 wt.% of Bi doping in SAC305 decreased the creep rate by 50%, and a 3 wt.% Bi doping in SAC305 also resulted in a decreased creep rate, to around half of its as-cast value. At higher aging temperatures of 75 °C and 125 °C, the effect of 2 to 3 wt.% Bi doping to SAC305 followed a similar trend but was more pronounced compared to the room-temperature aging. After aging for 90 days at 125 °C, the creep rate at 33 MPa increased tenfold for SAC305 and fivefold for SAC305+1 wt.% Bi compared to their as-cast conditions. Both SAC305+2 wt.% Bi and SAC305+3 wt.% Bi showed the opposite trend, with a creep rate at 33 MPa reducing to around 1/100 of their respective as-cast values. The different trends observed for samples with varying Bi contents clearly demonstrate the effect of Bi on the creep strength of SAC305.

## 4. Discussion

In this study, the sample preparation involved quenching of the mold that contained the solder materials in water to achieve a uniform microstructure. Using the heat transfer analysis described in [52], the cooling rate experienced by the samples was found to be between 5.2 °C/s and 5.6 °C/s. The JEDEC standard J-STD-020F recommends a cooling rate of less than 6 °C/s for solder reflow [45]. The analysis showed that the sample preparation method resulted in a cooling rate within the range specified by this JEDEC standard, although it was on the higher side. This cooling rate is also higher than those used in other studies of SAC+Bi solders and the traditional cooling rates recommended by most manufacturers (2–4 °C/s) [53]. 

In their as-cast conditions, all four alloys exhibited a pseudo-eutectic microstructure containing Ag_3_Sn and Cu_6_Sn_5_ IMCs and eutectic β-Sn embedded in a matrix of pro-eutectic β-Sn [45,50,54]. Bi precipitation was absent in the as-cast state for samples containing 2 wt.% Bi and 3 wt.% Bi, even though the room-temperature solubility of Bi in Sn is about 1.5 wt.%, according to the Sn–Bi phase diagram. Due to the high cooling rate experienced by the samples, non-equilibrium solidification was expected to have occurred in the as-cast samples to produce more of the β-Sn phase and less of the eutectic phase [42]. For the SAC+2 wt.% Bi and SAC+3 wt.% Bi samples, the high cooling rate also resulted in the excess Bi existing as supersaturated solid solutions instead of forming precipitations due to kinetic limitations. As the samples were aged at room temperature, Bi began to precipitate out from the Sn matrix, as revealed in the BSE–SEM images. While some studies reported that SAC305+3% Bi specimens prepared using a reflow cooling rate of 3 °C/s contained Bi precipitates in the as-reflowed condition [25,36], an observation similar to that of the present work was reported by Belyakov et. al., where SAC305–4% Bi solder joints prepared with a reflow cooling rate of 3 °C/s showed an absence of Bi precipitates in the as-reflowed condition [37]. In the same work, a progressive formation of Bi precipitations with room-temperature aging was reported, which is consistent with the observations in the present study. 

When the samples were subjected to aging, several microstructural changes occurred. For all compositions, coarsening of IMCs are expected, and the coarsening is more pronounced at elevated temperatures [45]. As the solubility of Bi in Sn increases with temperature, when the SAC305+2 wt.% Bi and SAC305+3 wt.% Bi samples were aged at elevated temperatures, Bi precipitates will be dissolved into the proeutectic Sn and diffuse through the Sn matrix to promote a more uniform distribution of Bi [55]. When the SAC305+2 wt.% Bi and SAC305+3 wt.% samples that were aged at 75 °C and 125 °C were removed from the ovens and allowed to cool to room temperature for nanoindentation and microstructure measurements, Bi precipitates reappeared. As shown in Figure 6, in SAC305+3 wt.% aged 125 °C, the Bi precipitates seem to be more uniformly and finely dispersed after increased aging duration, which can be attributed to the diffusion of Bi during aging. 

The observed hardness and creep strength of these water quenched samples can be correlated to their microstructures. In the as-cast states, excess Bi was dissolved in Sn as supersaturated solid solutions, and the higher hardness and lower creep strain rates observed for samples containing more Bi can be attributed to the effect of solid-solution strengthening [54]. For SAC305 and SAC305+1 wt.% Bi aged at any temperatures, a reduction in hardness and creep strength with aging was observed, and the decrease was more significant when these samples were aged at higher temperatures. This can be attributed to the coarsening of the Ag_3_Sn and Cu_6_Sn_5_ IMCs, a phenomenon well-documented for aged SAC solders [37,46,56,57,58]. It should be noted that since the cooling rate experienced by the samples was higher than the 2–4 °C/s cooling rate recommended by most manufacturers, the aging-induced changes in hardness and creep strength shown in Figure 7 and Figure 8 are relevant for specimens prepared using higher cooling rates. If slower cooling rates were used in the sample preparation, the quantitative results could have been different. On the other hand, enhanced hardness and creep strength were observed for the SAC305 containing 2 and 3 wt.% Bi, which is consistent with the literature. For example, the effect of 3.5 wt.% Bi addition in Sn0.7–0.5Cu on nanoindentation hardness and creep strength in the unaged condition was investigated by Liu et al. [59], where the authors reported an increase in hardness from 146.4 MPa to 315.9 MPa when 3.5% Bi was added to SAC0705. The same study also reported that, for the same creep strain rate, SAC0705 doped with 3.5% Bi had around a 60% higher stress value, indicating a significant increase in creep strength upon adding the Bi. In the present study, for SAC305+2 wt.% Bi and SAC305+ 3 wt.% Bi samples aged at room temperature, enhanced creep strength was observed, which was expected to be the resultant effect of multiple competing hardening and softening mechanisms. Since the water-quenched microstructures involved non-equilibrium solidification, the additional formation of Ag_3_Sn precipitates in the eutectic phase likely provided precipitation strengthening. The formation of Bi precipitates was expected to cause precipitation hardening, while the removal of Bi solute atoms from the Sn lattice led to softening with aging. The trend observed in Figure 7 and Figure 8 suggests that the hardening mechanisms had a greater effect compared to the softening mechanisms for these samples. Additionally, for SAC305+2 wt.% Bi and SAC305+ 3 wt.% Bi samples aged at elevated temperatures, the diffusion of Bi after being re-dissolved into the β-Sn matrix promoted a more uniform distribution of Bi. The Bi precipitates subsequently formed after removing the samples from the oven were more evenly and finely dispersed. While microstructure coarsening also occurred in these samples, the effect of precipitation strengthening was more pronounced, resulting in higher hardness and greater creep strength in these samples [60]. 

It is important to note that although the cooling rate of the sample preparation method in the present study was estimated to be within the range recommended by JEDEC, it was significantly above the reflow cooling rates commonly used by solder manufacturers (2–4 °C/s). The higher cooling rate used for this study is the fundamental reason for achieving a non-equilibrium solidification and all the subsequent results, including the aging-induced changes in microstructure and properties presented in this study, are a consequence of this non-equilibrium solidification, which potentially involved incomplete Sn–Ag eutectic reaction and an Sn lattice supersaturated with Bi in the as-solidified state. The aging-induced changes in microstructure and properties for solder joints prepared using slower cooling rates may be significantly different as slower cooling rates produce equilibrium microstructures with complete Sn–Ag eutectic reaction and precipitated Bi in an as-solidified condition. Therefore, the reflow process used for sample preparation can lead to variations in the microstructure and subsequent changes in properties. Inconsistencies regarding Bi precipitation in SAC+Bi alloys have been reported in the literature [37,38,39,40,41], likely due to variations in the sample preparation methods. A parametric study that involves varying cooling rates will be conducted in future work to further investigate the effect of Bi on microstructure and properties of SAC+Bi alloys. Future work will also investigate the effect of Bi on other properties, such as shear strength, of SAC–Bi alloys.

## 5. Conclusions

In this study, a detailed investigation was conducted to investigate the effect of 1, 2 and 3 wt.% Bi doping in SAC305 on the aging-induced changes in microstructure, hardness and creep strength for samples subjected to a high cooling rate. Sample preparation involved vacuum casting of micron sized samples of the four alloys suitable for nanoindentation using a glass mold-based technique, and the cooling rate experienced by the samples was between 5.2 °C/s and 5.6 °C/s. The specimens were subjected to nine different aging conditions consisting of three different aging temperatures of 25 °C, 75 °C, and 125 °C for each of the three aging durations of 30, 60, and 90 days. After aging was complete, the microstructures of the as-cast and aged samples were investigated under SEM-EDS to examine the phases present and the changes in microstructure induced by aging. Additionally, quasistatic and nanoDMA nanoindentation were used to determine the hardness and creep strength, respectively. Combining these experiments revealed a correlation between the microstructure and properties. Bi precipitates were absent in the as-cast states for all compositions, suggesting that non-equilibrium solidification of the samples and Bi precipitates formed progressively with aging in samples with 2 and 3 wt.% Bi. The Bi precipitates were more evenly and finely dispersed after aging at elevated temperatures due to the diffusion of Bi after they were re-dissolved in the β-Sn matrix. In the as-cast state, hardness and creep strength increased with increasing Bi content due to solid-solution strengthening. After aging at all temperatures, the hardness and creep strength were reduced in SAC305 and SAC305 doped with 1 wt.% Bi due to coarsening of the IMCs. After room-temperature aging, the hardness and creep strength of SAC305 doped with 2 and 3 wt.% Bi increased due to the dominant effect of strengthening by the formation of Bi precipitates. At elevated temperature, aging led to increased hardness and creep strength due to the formation of more uniformly and finely dispersed Bi precipitates. Results from this study suggest that when subjected to a high cooling rate, 2–3 wt.% of Bi can be beneficial for SAC305 to improve the hardness and creep strength. It should be noted that the conclusions in this study are relevant for specimens prepared using higher cooling rates. If slower cooling rates are used in sample preparation, the quantitative results could be different. Future work will consist of a parametric study varying the cooling rates and the effect of Bi on other properties of SAC–Bi alloys, including shear strength.

## Figures and Tables

**Table 1:** Aging conditions for isothermal storage.

Duration\Temperature	25 °C	75 °C	125 °C
0 days (as-cast)	X		
30 days	X	X	X
60 days	X	X	X
90 days	X	X	X

## Data Availability

Dataset available on request from the authors.
